# Heterogeneous tumor microenvironment in pancreatic ductal adenocarcinoma: An emerging role of single‐cell analysis

**DOI:** 10.1002/cam4.6407

**Published:** 2023-08-03

**Authors:** Sooin Byeon, Taymin du Toit‐Thompson, Josef Gillson, Anthony J. Gill, Jaswinder S. Samra, Anubhav Mittal, Sumit Sahni

**Affiliations:** ^1^ Northern Clinical School, Faculty of Medicine and Health University of Sydney Sydney New South Wales Australia; ^2^ Kolling Institute of Medical Research, University of Sydney Sydney New South Wales Australia; ^3^ Australian Pancreatic Centre Sydney New South Wales Australia; ^4^ Cancer Diagnosis and Pathology Group Kolling Institute of Medical Research St Leonards New South Wales Australia; ^5^ NSW Health Pathology, Department of Anatomical Pathology Royal North Shore Hospital St Leonards New South Wales Australia; ^6^ Upper GI Surgical Unit Royal North Shore Hospital and North Shore Private Hospital St Leonards New South Wales Australia; ^7^ The University of Notre Dame Australia Sydney New South Wales Australia

## Abstract

**Background:**

Pancreatic ductal adenocarcinoma (PDAC) is one of the deadliest malignancies in the world, for which the mortality is almost as high as the disease incidence and is predicted to be the second‐highest cause of cancer‐related deaths by 2030. These cancerous tumors consist of diversified gene expressions within the different cellular subpopulations that include neoplastic ductal cells, cancer‐associated fibroblasts, and immune cells, all of which collectively facilitate cellular heterogeneity in the PDAC tumor microenvironment (TME). Active intratumoral interaction within the cell populations in TME induces the proliferation of cancerous cells, accounting for tumorigenesis and rapid metastasis.

**Methods:**

This review will focus on novel findings uncovering PDAC heterogeneity in different cellular subpopulations using single‐cell RNA‐sequencing (scRNA‐seq) and other single‐cell analysis technologies. It will further explore the emerging role of single‐cell technologies in assessing the role of different subpopulations of neoplastic ductal cells, cancer‐associated fibroblasts, and immune cells in PDAC progression.

**Results and Conclusion:**

The application of scRNA‐seq in PDAC has started to unveil associations between disease progression and heterogeneity in pancreatic TME and could influence future PDAC treatment. Recent advances in scRNA‐seq have uncovered comprehensive analyses of heterogeneous ecosystems present within the TME. These emerging findings underpins further need for a more in‐depth understanding of intratumoral heterogeneity in the PDAC microenvironment.

## Graphical abstract

This review discusses recent advances in sequencing technologies at a single‐cell level, which are being employed for delineating the heterogeneity within different cellular subtypes in pancreatic tumors. As pancreatic tumors are highly heterogenous, further understanding of the differences within cellular subtypes will be crucial for better understanding the pathogenesis of this debilitating disease. It will be further useful to develop clinically relevant classification system for pancreatic tumors which could be used to stratify patients for personalized medicine approach using emerging targeted therapies.

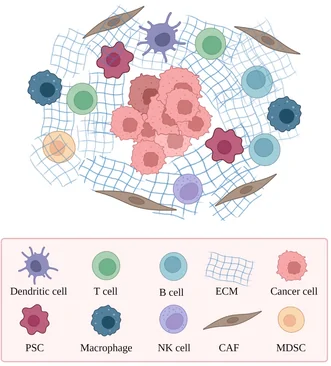

## 1 INTRODUCTION

Pancreatic cancer is one of the most lethal cancer types, with a 5‐year survival rate ranging from 2% to 9% globally.^1^ Pancreatic ductal adenocarcinoma (PDAC) is the most common form of pancreatic cancer that accounts for over 90% of all reported cases.^2^ The low survival rate of pancreatic cancer is attributed to several factors, the most significant of which is the advanced stage of the disease at diagnosis, with the majority of pancreatic cancer patients remaining asymptomatic until disease progression.^3^ Other risk factors for pancreatic cancer include family history, smoking, chronic pancreatitis, and sudden onset diabetes.^3^ Although surgical resection is the only treatment option that holds curative intent, only 15%–20% of the cases are resectable.^3^, ^4^ Of those patients that undergo resection, the majority of the patients are likely to have a recurrence and feature a 5‐year survival rate of up to 25%.^3^, ^5^ Currently, chemotherapy is the main systemic therapy available for PDAC patients, but chemoresistance is a major clinical problem leading to therapeutic failure.^6^ Hence, better understanding of complex tumor biology of pancreatic tumors is required to develop effective targeted therapies for this debilitating disease.

Genetic alterations in PDAC are typically seen in malignant neoplasms of the pancreas. These include the mutated oncogene; *Kirsten rat sarcoma viral oncogene homolog* (*KRAS)* which is most frequently altered in more than 90% of tumorigenesis, and three mutated tumor suppressor genes: *Cyclin‐dependent kinase inhibitor 2A (CDKN2A)*, *Tumor protein P53 (TP53)*, and *SMAD family member 4 (SMAD4)*.^7^ *CDKN2A* is the most frequently mutated tumor suppressor gene in PDAC, with inactivating mutation inhibiting the normal protein function of p16 (protein coded by *CDKN2A*) in cell cycle regulation in more than 90% of pancreatic cancer cases.^7^ These somatic mutations in PDAC are induced at different stages of pancreatic intraepithelial neoplasia (PanIN), which are noninvasive precursor lesions in the pancreas.^7^, ^8^ Mutated oncogenes of *KRAS* and inactivated *p16/CDKN2A* are known for their presence in almost all low‐grade PanINs, supported by similar observations in the development of PanINs in genetically engineered mouse models triggered by mutant *KRAS*.^8^, ^9^ Interestingly, *KRAS* codon 12 mutations are relatively more prevalent in higher grades of PanINs, with an increased levels of *KRAS* mutations with the PanIN grade.^10^ Similarly, an accumulation of tumor protein p53 was detected in the advanced stages of PanINs, which aligned with the previously identified abnormal *TP53* genes restricted to high‐grade lesions.^11^, ^12^ *SMAD4* inactivation is also seen in later stages of PanINs along with *TP53* gene abnormalities.^7^, ^12^ Molecular alterations in *TP53*, S*MAD4*, and *p16/CDKN2A* typically undergo loss‐of‐function mutations which are difficult to therapeutically target. There has been increasing availability of targeted therapies for PDAC patients that are guided by their mutational signatures. For instance, PDAC patients with homologous recombination deficiency, due to *BRCA1/2* mutations, are treated with platinum‐based compounds along with the PARP inhibitor, Olaparib, as maintenance therapy.^13^, ^14^ However, patients with *BRCA1/2* mutation carriers only account for up to 7% of all pancreatic cancer,^13^ and thus further subcategorization of PDAC based on transcriptomic techniques is a necessary endeavor to discern broader and more clinically significant subtypes, which will aid in the development of tailored therapeutic strategies for the treatment of PDAC patients.

## 2 TUMOR MICROENVIRONMENT OF PDAC

**FIGURE 1:**
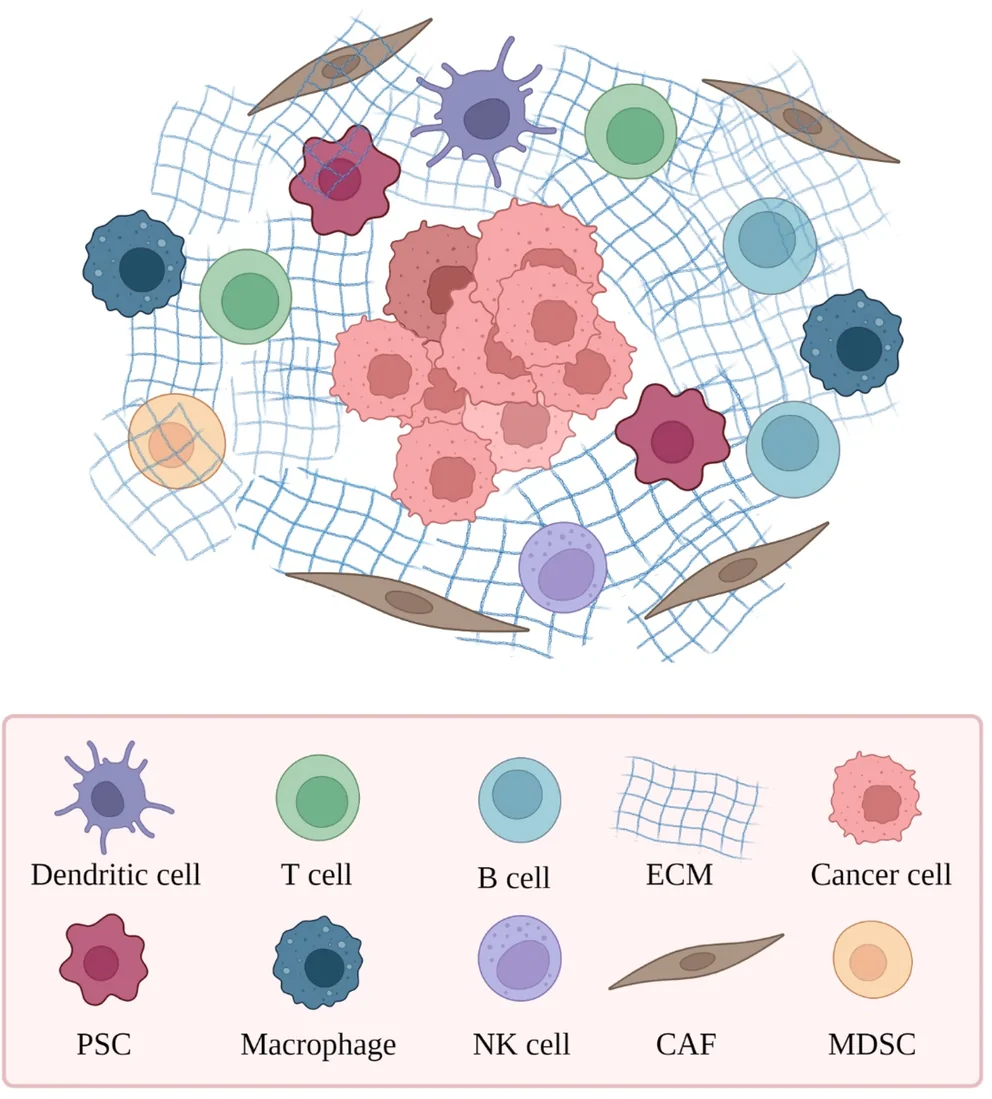
Various tumor‐associated cell populations dominant in PDAC TME. The tumor microenvironment (TME) of PDAC is composed of various cell types surrounded by dense extracellular matrix (ECM). Among different cell populations in PDAC TME, pancreatic stellate cells (PSCs), and cancer‐associated fibroblast (CAFs) are dominant cells that build a rigid barrier in favor of developing tumor‐promoting conditions, as well as inducing the proliferation of cancer cells. Other predominant cell types include macrophages, myeloid‐derived suppressor cell (MDSCs), regulatory T cells and natural killer (NK) cells. ^Created using Biorender.^

The PDAC microenvironment consists of several non‐cellular components, and various cell types, including cancer‐associated fibroblasts (CAFs), pancreatic stellate cells (PSCs), muscle fibroblasts, and immune cells (Figure 1).^15^ The PSCs and CAFs are dominant cell types in the dense stromal compartment contributing to an extracellular matrix in the tumor microenvironment (TME) and are responsible for producing a rigid barrier that favors tumor‐promoting conditions.^15^ CAFs play a pivotal role in tumorigenesis by reducing apoptosis and aiding cancer cells in their ability to migrate, proliferate, and remain viable.^16^ A co‐staining for well‐established CAF markers, such as alpha‐SMA and vimentin, showed unevenly distributed CAF markers through multiple cellular sources of CAFs which induce protumorigenic properties.^17^ Activated PSCs and mesenchymal stem cells (MSCs) are known for being major cellular sources for CAFs.^17^ Recent studies have further indicated that activated resident fibroblasts, endothelial cells, and fibrocytes, originating from other tumors, were also likely to be involved in tumor‐related processes and distinct functions in PDAC.^18^, ^19^

Other predominant cell types in the PDAC TME include regulatory T cells (Treg), myeloid‐derived suppressor cells (MDSCs), and macrophages, which are attributed to the formation of an immunosuppressive environment in PDAC due to their inhibition of certain CD8^+^T cells function in tumor recognition and clearance.^15^ The invasion of MDSCs and the upregulation of PD‐L1 through IFN‐γ are characteristic of approximately 50% of PDAC tumors.^15^ Additionally, in more advanced tumors it has been found that Tregs and effector T cells (Teffs) contribute to the immunosuppressive environment of the TME by inhibiting normal T cell immunoprotection.^15^, ^20^ It has been established that there is an above average levels of MDSCs in the peripheral blood of resected PDAC patients, and an accumulation of both MDSCs and macrophages in murine PDAC tumors.^20^ When targeting these cells with phosphodiesterase‐5 inhibitor (sildenafil), it resulted in prolonged survival in PDAC murine models with evidence of restored anticancer immune responses, reversing the immunosuppressive TME.^20^

The microenvironment in PDAC is highly heterogeneous, with various cell types that constitute the TME. Furthermore, evidence from recent studies suggests that the same cell type could have different genotypic and phenotypic characteristics with more specialized functions within the PDAC TME.^21^, ^22^ Thus, detailed investigations using emerging technologies, such as single‐cell RNA‐sequencing (scRNA‐seq) are required to further understand the role of the complex TME in PDAC progression.

## 3 BULK TISSUE TRANSCRIPTOMICS IN PDAC TUMORAL HETEROGENEITY

### 3.1 Classification of PDAC subtypes

**FIGURE 2:**
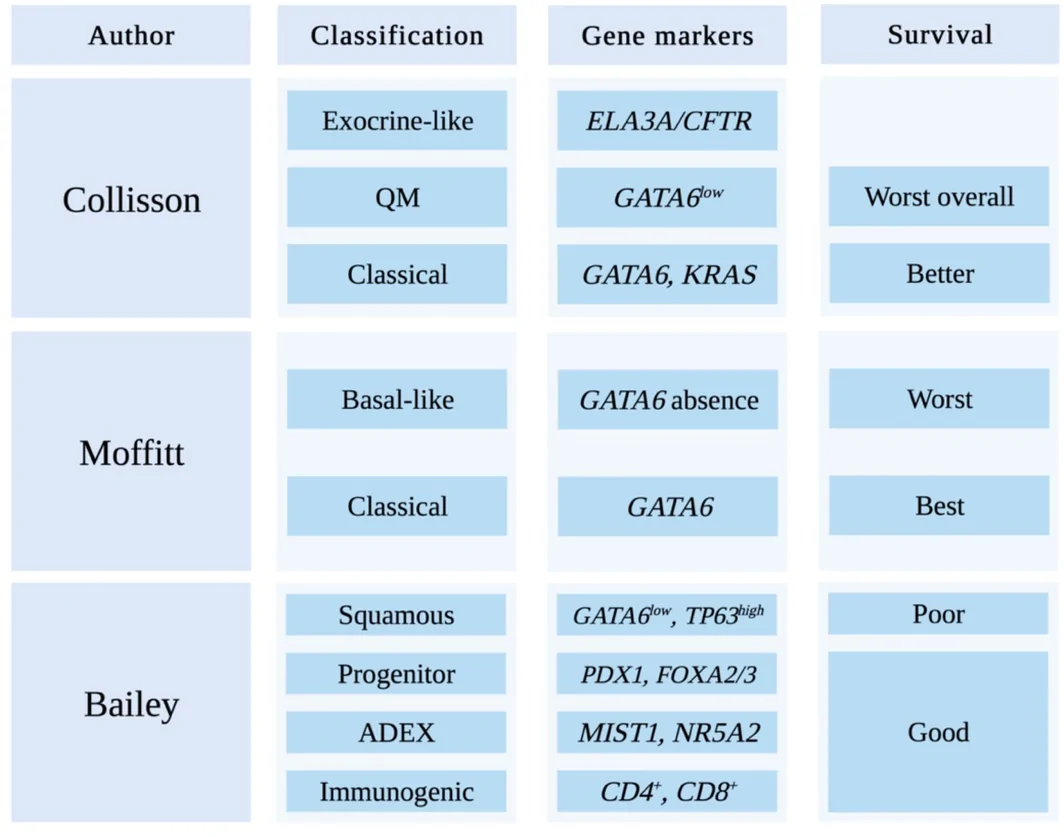
Different classifications of PDAC subtypes by genomic analysis. There are generally three classifications of PDAC subtypes by genotypic characterization. Collisson et al. defined three subtypes (exocrine‐like, quasimesenchymal (QM), and classical) and identified associated gene markers to each subtype. Both QM and classical subtypes are associated with *GATA6* but with contrasting expression levels. Moffitt et al. proposed a binary classification of PDAC, basal‐like, and classical subtypes. In contrast to the classical subtype, basal‐like is characterized by the absence of the *GATA6* gene marker. Bailey et al. defined four subtypes of PDAC by phenotypic features (squamous, progenitor, aberrantly differentiated endocrine exocrine (ADEX), and immunogenic). Except for the squamous subgroup which is low in *GATA6* and high in tumor‐promoting *TP63*, the other three subgroups are associated with a good survival outcome. ^Created using Biorender.^

The variety of cell types found throughout the TME in PDAC contributes to the complexity of determining prognosis. This is compounded by the fact that these cells are often genotypically heterogeneous. Several studies have analyzed pancreatic tumors using bulk tissue techniques to suggest broad classifications of PDAC and identified specific gene expressions which characterize different subgroups of PDAC (Figure 2). Collisson et al.^23^ defined three molecular subtypes of PDAC, namely, classical, quasimesenchymal (QM), and exocrine‐like. Classical and QM subtypes were associated with *GATA binding protein‐6* (*GATA6*), involved in normal development and cancer pathophysiology. *GATA6* was found highly expressed in classical subtype tumors and cell lines, whereas QM cell lines and tumors had a lower expression level of *GATA6*.^23^ RNA interference of *GATA6* on colony formation in the classical‐PDAC cell lines resulted in impaired anchorage‐independent growth, which was absent in the QM cell lines.^23^ This indicated a subtype‐specific role of the GATA6 transcription factor in PDAC progression. The ability to differentiate between two subtypes of PDAC cells and their contrasting use for the protein GATA6 not only enforced the understanding that these subtypes are molecularly different but could also benefit the development of tailored treatment regimens of patients with different targeted therapies.

In 2015, Moffitt et al.^24^ introduced a binary classification of PDAC characterized by basal‐like and classical subtypes. Further, Bailey et al.^25^ proposed four PDAC subclasses: squamous, pancreatic progenitor, immunogenic, and aberrantly differentiated endocrine exocrine (ADEX), based on the differential expression of transcriptional networks in PDAC. Squamous subtype had the worst prognosis of all, and these tumors were found to be enriched for *TP53* and *KDM6A* mutations, which were characterized by elevated levels of *TP63∆N* and its target genes.^25^ This leads to the regulation of epithelial cell plasticity, tumorigenicity, and epithelial to mesenchymal transition.^25^ Pancreatic progenitor and ADEX subtypes identified coexpressions of apomucins, specifically *MUC5AC* and *MUC1* types, which indicated the presence of pancreaticobiliary subtype of intraductal papillary mucinous neoplasm (IPMN).^25^ There is a substantial overlap in these three main classification systems, and additional studies have continued to contribute their interpretations of PDAC subtype classification based on bulk transcriptomics analysis.^26^ These classification systems aim to decipher and clarify the diverse heterogeneous presentation of PDAC in the clinical setting and assist researchers to develop targeted therapies. However, these classification methods only relied on differential gene expression between the bulk tumors and did not take into consideration of heterogeneous populations in cellular subclasses in PDAC TME. Recently, studies have started to uncover these complex cellular subclasses by utilizing advances in molecular analysis at the single‐cell level.

### 3.2 Coexistence of different genotypes within classical biopsy‐derived pancreatic cancer organoids

Although bulk RNA‐sequencing has enabled genotypic classifications of PDAC tumors, this approach may not be adequate to delineate heterogeneous cellular subpopulations in PDAC TME, with growing evidence that more than one genotypic characteristic can be portrayed by different cell types at the single‐cell level within a tumor. Juiz et al.^22^ conducted a study on epithelial cells from six different PDAC organoids, that were initially defined as “classical” using bulk transcriptomics analysis, to demonstrate a significant heterogeneity within these specimens. Four major cell clusters (C0–C3) were identified by scRNA‐seq, in different proportions in all tumors, where cluster C1 was characterized by highly expressed basal gene markers that significantly corresponded to basal components.^22^ Genomic classifications by Bailey et al.^25^ and Collisson et al.^23^ applied to single‐cell analysis had shown that cluster C1 was high in squamous, QM, and basal‐like subtypes, whereas both classical and immunogenic signatures were at the lowest levels.^22^ Similarly, Krieger et al.^21^ also showed coexistence of “classical” and “basal‐like” state in PDAC organoids by single‐cell transcriptome analysis. These observations supported the coexistence of multiple genotypes within a single tumor and the complex heterogeneity of PDAC tumors. Therefore, scRNA‐seq could be used to further classify specific molecular and cellular differences between patient tumors.

One of the biggest problems in PDAC treatment has been the lack of patient response to various chemotherapeutic strategies, which could be a result of intratumoral heterogeneity in PDAC and the limited specific targeting of certain tumor subtypes. As bulk sequencing only measures an average gene expression of cell populations, profiling of individual cells cannot be achieved for more in‐depth analysis of heterogeneous microenvironment. This would be highly beneficial in the development of personalized targeted therapies in future. Thus, the application of scRNA‐seq can be used as a means of characterization of PDAC to aid with patient stratification and tailored treatment strategies in future.

## 4 SINGLE‐CELL ANALYSIS IN PRIMARY PDAC TUMORS AND METASTATIC LESIONS

### 4.1 Technologies in single‐cell analysis

While bulk sequencing can help determine the average expression level of cells inhabiting the PDAC TME and assist prognosis, novel single‐cell transcriptomic techniques are required to decipher the genotypical diversity of cells in TME. Single‐cell analysis characterizes gene expression and mutation data of individual cells in tissues at the single‐cell resolution, which can thereby identify heterogeneous cell populations regardless of their abundance within the TME.^27^ A recent meta‐analysis of scRNA‐seq data identified 17 cell categories in PDAC TME, which detected the loss of anticancer immune response in natural killer cells and CD8^+^ T cells manifested by exhaustion‐related or immune checkpoint blockade resistance markers, affecting the prognostic potential in the overall survival of PDAC patients.^28^

In a recent study, single‐cell mass cytometry was utilized to identify three immune‐based subtypes of PDAC tumors based on the expression of programmed cell death protein 1 (PD‐1) and CD8 in tumor‐infiltrating immune cells and their associated immune pathways.^29^ The subtype S2, exhibiting low PD‐1 and CD8 expression, was associated with worse prognosis and lower immune infiltrate when compared with the S1 subtype with high PD‐1 and CD8 expression.^29^ The enriched ECM interactions in the S2 subtype were attributed to the lower immune infiltrate.^29^ This classification could be used as a potential biomarker for PDAC prognosis.

Integrating transcriptomic profiling and T‐cell receptor (TCR) sequencing of primary tumors in PDAC revealed heterogeneous tumor‐infiltrating lymphocyte^30^ populations that contained seven CD8^+^ and five CD4^+^ TIL cell states.^31^ The analysis of ex vivo culture of tumor samples showed selective expansion of particular Teffs that have initially also taken place in situ.^31^ Given that T cells could facilitate transitioning between different functional states, trajectory mapping identified their relationships with different TIL states. For example, CD8‐GZMK was suggested to be an intermediate cell state in transitioning between the CD8‐GZMB/PRF1 and CD8‐CXCL13 cell states that were identified as cytotoxic cell states.^31^ Although *EOMES* expression in CD8‐GZMK induced T‐cell exhaustion (i.e., loss of the ability to kill certain cells), the lack of checkpoint molecule expression and exhaustion markers identified may indicate a transitional cell state with dysfunctional features of T cells to some extent.^31^ Understanding TIL cell states at the single‐cell resolution differentially expressed in PDAC reinforced the heterogeneity of cell compartments within TIL populations and contributed to a foundation to implement currently unexplored immunotherapeutic treatments.

Additionally, based on a hostile relationship between tumor‐infiltrating T cells and malignant ductal cells enriched in PDAC, receptor–ligand interactions were analyzed at the single‐cell resolution to investigate T cell and tumor cell interactions.^32^ The cross‐species scRNA‐seq analysis in human and mouse PDAC tissues found that chemokine CCL5 acted as a specific ligand secreted by T cells, and the receptors SDC1 and SDC4 were highly expressed in malignant ductal cells in PDAC.^32^ This demonstrated tumor‐promoting features of CCL5‐SDC1/4 interactions activated in PDAC, which could potentially be used as prognostic biomarkers to track tumor progression.

Single‐cell transcriptomics in low‐grade IPMNs detected a small population of proliferating epithelial cells enriched with oncogenes, while the majority being in a low proliferative state.^27^ The gradual depletion of T lymphocytes, that make up approximately 1%–2% of cell populations, was observed when transitioning from low‐ and high‐grade IPMN samples to fully transformed PDAC tumors.^27^ This contributed to some changes to microenvironmental components at the single‐cell levels. Through single‐cell digital microdissection of IPMN and PDAC, rare B‐cell populations were observed only in IPMNs (low‐ and high‐grade) and absent in PDAC lesions.^27^ Moreover, CD4^+^ and CD8^+^ T cells were found in higher numbers in both low‐ and high‐grade IPMNs, in contrast to a much lower number of these cells in PDAC tumors enriched with MDSCs.^27^

Furthermore, single‐cell multiomics sequencing methods make it more feasible to assess the epigenomic abnormality in PDAC, which had been challenging due to the high interpatient heterogeneity. Single‐cell multiomics sequencing has allowed researchers to identify valuable information concerning the DNA methylome, chromatin accessibility, copy number variation, and ploidy of individual cells in PDAC patients.^33^ For instance, Fan et al.^33^ applied single‐cell Chromatin Overall Omic‐scale Landscape Sequencing (COOL‐seq) and discovered that cancer cells in primary tumors were enriched for DNA demethylation in heterochromatin regions, and the RNA expression in gene body regions of cancer cells was negatively correlated with promoter DNA methylations. Alternatively, normal epithelial cells from primary tumors produced a significant proportion of euploid genomes and clusters with those from adjacent normal tissues.^33^ Some normal epithelial cells in PDAC clustered together with adjacent normal cells, and these clustered cells were similar to type 1 ductal cells.^33^ Notably, type 1 ductal cells are known to have gene expression pattern similar to normal ductal cells.^33^, ^34^ The remaining non‐clustered normal epithelial cells in PDAC showed cancer‐like features in gene expression, representing a diversity of TME of PDAC.^33^

While scRNA‐seq technologies have significantly advanced our understanding of PDAC heterogeneity, these technologies suffer in their applicability given the high intrinsic nuclease activity and the dense desmoplastic stroma in PDAC.^30^, ^35^ To overcome these issues and current requirements of scRNA‐seq, including the necessity for single‐cell suspensions to be prepared from fresh samples, an alternative method termed single‐nucleus RNA‐sequencing (snRNA‐seq) was introduced. Unlike scRNA‐seq, snRNA‐seq is applicable for frozen or hard‐to‐dissociate samples, allows for multiplexed analysis of longitudinal samples from a single patient, and has shown better recovery for both malignant and stromal tissues following processing.^30^, ^36^ Combined with whole‐transcriptome digital spatial profiling, the snRNA‐seq analysis allowed researchers to identify an integrated spatial and cellular profile of three multicellular communities with distinct features of malignant, stromal, and immune subtypes: classical, squamoid‐basaloid, and treatment enriched.^30^ A treatment enriched community in post‐chemotherapy treated patients was abundant in neurotropic CAF programs and CD8^+^ T cells.^30^ Classical community was involved in myofibroblastic progenitor and adhesive CAF programs, while squamoid‐basaloid community had their malignant programs with higher level of epithelial and immune cells.^30^

Additional snRNA‐seq technologies such as single nuclei RNA‐seq (sNuc‐seq) use droplet microfluidics to encapsulate single nuclei with mRNA capture beads, allowing the transcriptomes of thousands of nuclei to be analyzed to reveal distinct cell types.^37^ Uncovering the dynamics of pancreas development, the unique interactions of immune cells, and the differences in cellular composition compared to the adult pancreas has been possible due to sNuc‐seq data that has been generated from neonatal tissue.^37^ Therefore, use of this technique in PDAC could aid in the development of tailored therapeutic strategies for the treatment of early versus last stage patients.

Neither sequencing technologies are a replacement for the other and researchers must discern when scRNA‐seq and/or snRNA‐seq techniques are necessary for their research question. When fresh tissue is available, massive parallel analysis scRNA‐seq methods such as Droplet‐seq (Drop‐seq) can be utilized, while when stores of frozen tissue are available high‐throughput snRNA‐seq methods such as sNuc‐seq can be implemented.^38^ Additionally, methods such as DroNc‐seq (massively parallel sNuc‐seq with droplet technology) combine the benefits of sNuc‐seq and Drop‐seq to perform cost‐effective high‐throughput profiling of nuclei.^38^ These emerging technologies will allow a more robust and translatable understanding of the genotypical diversity of cells in the PDAC TME which will further augment innovative strategies of exploiting PDAC tumors therapeutically.

### 4.2 Distinct cell types in PDAC tumor microenvironment

Recent advances in single‐cell analysis have revealed intratumoral heterogeneity in the PDAC TME. The PDAC TME consists of diverse malignant and stromal cell types, including type 1/2 ductal, acinar, endocrine, endothelial, fibroblasts, stellate, and T and B cells.^34^ Gene markers associated with these cell types have been identified to be specific to each cell cluster in PDAC, such as *KRT19* and *TFF2* for ductal cells, *LUM* for fibroblasts, and *AIF1* for macrophages.^34^

#### 4.2.1 Ductal cells in PDAC tumors

Ductal cells from primary PDAC tumors were considered one of the main cell populations that constitute PDAC TME and were found enriched for ductal markers, such as *MMP7*, *TSPAN8*, *SOX9*, *KRT19*, and *LCN2*.^34^ Changes in cellular compositions of the TME in PDAC were suggested by a contrasting trend in ductal cells that were decreased during PDAC tumor stage progression as opposed to an increased level of immune cells.^39^ Ductal cells were dominant in the early stages of PDAC, accounting for 44.88% of cellular structures, whereas only 17.06% of cells were identified as immune cells.^39^ As the staging of PDAC progressed the proportion of immune cells increased gradually, to 36.15% in stage II and 43.14% in stage III, which could be due to the need for a greater immune response against tumor development.^39^ Two ductal cell subtypes were identified and distinguished by their malignant status and functional features in PDAC based on their gene expression patterns using scRNA‐seq analysis.^34^ Type 1 ductal cells were dominant in a benign group of pancreatic tumors, making up approximately 50% of their cellular composition, which was supported by the expression of characteristically benign genes found in normal epithelial cells.^34^ Thus, type 1 ductal cells were more reserved in normal pancreatic functions, such as digestion, pancreatic secretion, and bicarbonate support, which was further validated by *AMBP*‐positive cells with normal cell features in type 1 ductal cells in PDAC and normal pancreas.^34^ However, approximately 85% of the upregulated genes found in type 1 are also found in type 2 ductal cells, further indicating the abnormal state of type 1 ductal cells compared to healthy pancreas cells.^34^

Type 2 ductal cells are characterized as malignant, given that their incidence was 4.3‐fold higher in PDAC samples compared with type 1 ductal cells, along with the presence of PDAC gene markers of poor prognosis which included *CEACAM1/5/6* and *KRT19*.^34^ *MUC1*‐ and *FXYD3*‐positive cells were only present in PDAC also supported malignant gene expression profiles of type 2 ductal cells.^34^

Copy number variation in type 2 ductal cells in PDAC was also observed at higher levels compared with type 1 ductal cells, which resulted in the loss of normal functioning pancreas and distinguished type 2 ductal cells from other major cell populations.^34^ Comparatively, genes associated with cell proliferation and migration were identified to be upregulated in type 2 ductal cells, which was also enriched in later stages of PDAC.^34^, ^39^ Additionally, other cancer‐related functions, such as chemokine receptor binding, R‐SMAD binding, cytokine activity, adhesion, and chemokine activity, were identified in type 2 ductal cells in the advanced stages of PDAC.^39^ All these findings demonstrated the malignant state of type 2 ductal cells, as opposed to the non‐cancerous trait of type 1 ductal cells.^39^ Furthermore, at the single‐cell resolution, ductal cells persisting during later stages of PDAC were associated with cancer stem cell (CSC) related genes and mesenchymal markers, further illustrating the heterogeneity of the PDAC TME during tumor progression.^39^

#### 4.2.2 Stromal cancer‐associated fibroblast (CAF)

CAF is a broad term referring to the cells within a tumor that carry similar characteristics to myofibroblasts.^40^ These cells play an important role in supporting cancer cells during tumor development and progression.^40^ The fibroblast‐enriched fraction of human PDAC was associated with the formation of two distinct CAF subclusters, myofibroblastic CAFs (myCAFs) and inflammatory CAFs (iCAFs) within fibroblasts.^41^ Although common fibroblast markers such as *COL1A1*, *FAP*, and the mesenchymal cell marker, *VIM*, were found in both subtypes, there were specific gene signatures identified for each subcluster.^41^ This illustrated the unique function of the two subpopulations during tumor progression in PDAC. Given the high levels of alpha‐smooth muscle actin (ACTA2) in myCAFs, the muscle contractile proteins, such as TAGLN, MYL9, and TPM1/2, were enriched for the upregulation of smooth muscle contraction, focal adhesion, and collagen formation.^41^ In contrast, iCAF markers included *CFD*, *LMNA*, and *DPT*, which target inflammatory pathways such as IFNγ response, TNF/NF‐κB, and IL2/STAT5.^41^ Such interactions from both iCAFs and myCAFs are crucial for supporting tumor progression.

Recent scRNA‐seq of murine PDAC tumors has identified an additional CAF subtype termed antigen‐presenting CAFs (apCAFs) due to their expression of MHC class II‐related genes and ability to activate CD4^+^ T cells in an antigen‐specific manner.^41^ These CAFs are present in human PDAC and expresses *HLA* encoding MHC class II chains and *CD74* encoding SLPI, which is a prominent apCAF marker in mouse PDAC. While the expression profile of apCAFs in murine PDAC has been described, the apCAF gene signatures in human PDAC is still required to be elucidated.

A new subset of CAFs in the PDAC microenvironment, complement‐secreting CAFs (csCAFs) was identified, with a strong expression of csCAF‐related genes, which were identified by weighted gene coexpression network analysis (WGCNA).^39^ In tumor specimens from PDAC patients, the number of csCAFs were significantly high in stage I, followed by a gradual decrease in relative counts of csCAFs with tumor progression to Stage II and III, suggesting a tumor‐suppressive feature of csCAFs.^39^

Single‐cell analysis utilizing mass cytometry of murine and human healthy tissues and tumors has revealed two distinct pancreatic fibroblast lineages based on their expression of the transforming growth factor β receptor (TGF‐βR) coreceptor, CD105.^42^ It was found that CD105‐positive pancreatic fibroblasts supported in vivo tumor growth, while CD105‐negative fibroblasts strongly suppressed tumor growth depending on functional adaptive immunity. Following mass cytometry analysis, the authors reanalyzed available scRNA‐seq data to confirm that both CD105‐positive and CD105‐negative fibroblasts can acquire iCAF and myCAF characteristics, while apCAF gene expression is primarily limited to the CD105‐negative subset.^41^, ^42^ It could be proposed that suppressing the activity of the tumor permissive CD105‐positive fibroblasts and promoting the tumor‐suppressive properties of CD105‐negative fibroblasts it may be possible to mitigate PDAC tumor progression and aide in treatment. Hence, single‐cell analysis has allowed for critical fibroblast heterocellular interactions to be uncovered that may be exploited for future therapeutics.

Another new subset of CAF with a highly activated metabolic state (meCAFs) was found in loose‐type PDAC compared with dense‐type PDAC.^43^ Wang et al.^43^ identified that infiltrating CD8^+^ cells were increased in loose‐type PDAC with an enhanced of cytotoxic ability. Loose‐type PDAC were characterized for matrix‐poor TME, so‐called “immune‐hot” tumors, indicating more susceptibility to metastasis and more abundant immune cells in TME.^43^ Loose‐type PDAC was featured abundance of meCAFs compared with dense‐type PDAC, and the presence of PLA2G2A and CRABP2 meCAF markers was distinct in loose‐type PDAC.^43^ Given that the presence of meCAF markers in the tumor were related to poor overall survival of PDAC patients, this could suggest that the presence of meCAFs in tumor specimens could be a prognostic indicator.^43^ The authors further evaluated the efficacy of PD‐L1 antibody in 17 patients with high levels of meCAFs and a better response to immunotherapy was achieved in those patients treated with chemotherapy combined with PD‐L1 antibody compared to PD‐L1 antibody alone.^43^ These results demonstrated the potential utility of tailored therapeutic strategy arising from single‐cell analysis.

#### 4.2.3 Immune cells

Suppressive immune cells in the PDAC TME are comprised of heterogeneous compartments with dendritic cells (DC) and immature myeloid cells and macrophages. DCs promote antitumor functions in cytotoxic T cells to suppress tumor growth. In contrast, immature myeloid cells and tumor‐associated macrophages (TAMs) act as leading factors in tumor progression and immune evasion.^44^

Rodriguez et al.^44^ had reanalyzed the scRNA‐seq data published by Peng et al.^34^ to further delineate myeloid cells in PDAC.^44^ Only the cells within myeloid clusters were selected for reclustering and five clusters were identified.^44^ The most abundant myeloid cells in PDAC TME were monocyte‐derived macrophages (moMac), monocyte‐derived dendritic cells (moDC), and monocytes.^44^ Proliferating monocytes, representing less than 5% in PDAC, were characterized by carrying the *MKI67* proliferation marker and other cell cycle‐associated genes.^44^ Tissue‐resident macrophage (trMac) were mostly observed in normal pancreatic tissues, unlike the rest of the four clusters.^44^ The higher abundance of moMac in patient tumor was correlated with a shorter survival, while a longer survival was observed in patients who had enrichment of moDC.^44^ Hence, single‐cell analysis of myeloid cells in PDAC could characterize their compartments in tumors, which could be further used as a predictor for patient prognosis.

TAMs are the most abundant tumor‐infiltrating immune cells in PDAC, consisting of two subgroups: immune‐stimulatory macrophages (M1) and immune‐regulatory macrophages (M2).^45^ Increased levels of CD68^+^ macrophages and CD163^+^ M2 in human PDAC tissues resulted in a poor overall survival outcome.^45^ Further, the expression levels of CD47 were shown to correlate with CD68^+^ macrophages, but not with CD163^+^ M2.^45^ Based on the efficacy of anti‐CD47 treatment to restrain tumor growth in PDAC, a decrease in monocyte/macrophage populations was noted, whereas the lymphoid populations were increased in mouse models.^45^ CD47 blockade demonstrated changes in the intratumoral macrophage compartment at the single‐cell resolution using scRNA‐seq. Among five macrophage clusters (C1‐5) identified, C3, 4, and 5 were significantly more abundant compared to the C1 and C2. Notably, cluster 3 was the most enriched for *nitric oxide synthase 2* (*NOS2*) and was responsible for regulating inflammatory reactions.^45^ The inhibition of C1 and 2 resulted in a decreased expression level of the *macrophage mannose receptor 1* (*MRC1*), which reduces anti‐inflammatory responses and immunosuppression processes.^45^ Furthermore, Chen et al.^46^ performed scRNA‐seq and revealed M2 macrophage showed a higher expression of immune marker, SPP1, in PDAC compared to those in normal pancreatic tissues. Using multicolor immunohistochemistry (IHC) staining further analysis revealed abundant chemokines and ligands in M2 macrophage, which induced tumor development in PDAC.^46^ This suggested SPP1 is a potential prognostic biomarker in PDAC, considering its abundance in type 2 ductal cells and M2 macrophage, both of which were involved in tumor‐related characteristics. Overall, single‐cell analysis has helped uncover immune‐related signatures for PDAC and allowed us to start delineating cellular interactions between different cell types during tumor progression and provide a framework to approach novel treatments.

Steele et al.^47^ uncovered a heterogeneous immune landscape in PDAC through multimodal mapping using a combination of CyTOF, scRNA‐seq, and multiplex IHC. CD8^+^ T cells and NK cells were found abundant in the PDAC microenvironment, acting as pro‐inflammatory cells. By unbiased clustering, tumor‐infiltrating CD8^+^ T cells were clustered into six populations of CD8A‐expressing T cells in PDAC samples and adjacent/normal samples, which were featured by the expression of dominant genes per cluster: two populations of effector CD8^+^ T cells (T_eff_) that expresses *PRF1* and *GZMB*; one population of memory/precursor CD8^+^ T cells (T_mem/pec_) expressing CCR6; and two other populations of exhausted CD8^+^ T cells (T_ex_) with the expression of *EOMES*, *GZMK*, and *TIGIT*. In tumor‐infiltrating CD8^+^ T cells, T_ex_ cells, and T_mem_ cells were relatively increased with the enrichment of immune checkpoint receptor, *TIGIT* compared to adjacent/normal CD8^+^ T cells. There was also relative decrease in the T_eff_ levels in tumor infiltrating CD8^+^ T cells.

Three clusters of NK cell subpopulations were also observed in PDAC specimens, which were characterized by gene expression markers of antigen presentation (*HLA‐DRA*), cytolytic activity (*PRF1* and *GZMB*), and chemokines/chemokine receptors (*CCL3* and *IL7R*). Tumor‐infiltrating NK cells had a differential gene signature in advanced disease samples, with increased activation markers such as *GZMA* and increased expression of two immune checkpoint genes, *TIGIT* and *HAVCR2*. Further, transcriptional profiling of CD4^+^ T cells, revealed 13 distinct populations, but a number of these clusters seemed to merge together. *TIGIT* was highly enriched in Tregs in particular, along with a sparse expression in other clusters.

## 5 RECENT EMERGING TECHNOLOGIES

New single‐cell technology, such as CosMx Spatial Molecular Imaging (SMI),^36^ has recently been introduced as a high‐plex profiling method to detect RNA and proteins from formalin‐fixed paraffin‐embedded (FFPE) and fresh frozen samples at a single‐cell resolution with intact spatial annotations.^48^ Unlike scRNA‐seq which requires tissue dissociation and cell isolation leading to the loss of spatial information, CosMx SMI demonstrates the in situ measurements of RNA and proteins on intact FFPE and fresh frozen samples by single‐molecule barcoding [49]. This generates high sensitivity and very low error rate with a completely automated system, which offsets the limitation of the scRNA‐seq method.

## 6 CONCLUSION

Single‐cell sequencing in PDAC has led to identifying intra‐tumoral heterogeneity within TME (Table 1). Furthermore, snRNA‐seq has made single‐cell sequencing more accessible by utilizing frozen samples, and it has advanced cellular profiling in PDAC TME. While this review has covered recent advances in the field of single‐cell analysis and identified the significance of investigating intratumoral heterogeneity within PDAC TME, further studies are necessary to discern the complex microenvironment of PDAC using constantly emerging latest technologies. This will be critical for our understanding of intricate TME in PDAC and will further assist in the development of personalized medicine based targeted therapies for PDAC patients.

**TABLE 1:** Summary of major studies delineating PDAC heterogeneity using single‐cell analysis technologies.

Authors	Findings	Technologies used
Tang et al.^28^	Seventeen cell categories in PDAC TME. Loss of anticancer immune response in NK and CD8^+^ T cells.	scRNA‐seq dataset and bulk sequencing data for bioinformatics analysis
Wang et al.^29^	Three Immune‐based subtypes based on PD‐1 and CD8 expression in tumor‐infiltrating immune cells. S2 cluster manifested by low PD‐1 and CD8 as opposed to cluster S1. S2 cluster had worse prognosis and lower immune infiltration.	IHC, single‐cell mass cytometry
Schalck et al.^31^	Heterogeneous TIL populations in PDAC. Seven CD8^+^ and five CD4^+^ TILs. CD8‐GZMK acting as an intermediate cell state transitioning as cytotoxic cell state. CD8‐GZMK lacking T‐cell exhaustion markers.	scRNA‐seq, TCR sequencing, pseudotime trajectory analysis
Chen et al.^32^	Chemokine CCL5 secreted by T cells, acting as a specific ligand. Receptors SDC1/4 abundant in malignant ductal cells in PDAC.	scRNA‐seq, inferCNV analysis
Bernard et al.^27^	Small population of proliferating epithelial cells in low‐stage IPMN. T lymphocytes depletion from IPMN to PDAC. Rare B‐cells only in IPMNs. CD4^+^ and CD8^+^ T cells more abundant in IPMNs.	scRNA‐seq, single‐cell digital microdissection
Fan et al.^33^	Enriched for DNA demethylation in cancer cells of PDAC. Negative correlation between RNA expression and promoter DNA methylations. Euploid genomes and clusters abundant in epithelial cells in PDAC.	Single‐cell multiomics sequencing, scCOOL‐seq
Hwang et al.^30^	Refined molecular classifications of PDAC with three distinct multicellular communities; classical, squamoid‐basaloid, and treatment enriched.	snRNA‐seq, whole‐transcriptome digital spatial profiling
Peng et al.^34^	Benign trait of Type 1 ductal cells with normal pancreatic functions, as opposed to malignant state of type 2 ductal cells. Abnormal state of type 1 ductal cells with upregulated malignant genes.	scRNA‐seq, cell trajectory analysis
Chen et al.^39^	Gradual increase of immune cells in later stages of PDAC, as opposed to ductal cells. Upregulation of genes related to cancer‐related functions in type 2 ductal cells in later stages of PDAC. Gradual decrease of csCAFs as tumor progresses.	scRNA‐seq, WGCNA, RNA‐FISH
Elyada et al.^41^	apCAFs in murine PDAC manifested by MHC class II‐related genes and CD4^+^ T cells activation.	scRNA‐seq
Hutton et al., Elyada et al.^41^, ^42^	Suppression of tumor growth in vivo with CD105‐negative fibroblasts, as opposed to the presence of CD105‐positive fibroblasts. CD105 fibroblasts can acquire both iCAF and myCAF characteristics. apCAF gene expression limited to CD105‐negative subset.	Single‐cell analysis in mass cytometry, scRNA‐seq, bulk RNA‐seq
Wang et al.^43^	meCAFs abundant in loose‐type PDAC, where high in infiltrating CD8^+^ cells with cytotoxic ability. meCAFs could be used as prognostic biomarker. More immune cells in loose‐type PDAC and could be used as a biomarker for immunotherapy response in PDAC.	scRNA‐seq
Rodriguez et al.^44^	Five myeloid were clusters identified. Patients with moMac‐enriched tumors had poor prognosis.	Bulk sequencing, scRNA‐seq analysis
Pan et al.^45^	Poor patient survival when abundance of CD68^+^ macrophages and CD163^+^ M2 were increased in tumors. CD47 blockade leads to changes in intratumoral macrophage compartment.	IHC, scRNA‐seq
Chen et al.^46^	SPP1 is an immune marker which is abundant in type 2 ductal cells and M2 macrophage in PDAC.	scRNA‐seq, IHC
Steele et al.^47^	Six populations of tumor infiltrating CD8^+^ T cells were identified in PDAC tumors. Three distinct clusters of NK cells were identified in PDAC tumors. Thirteen populations of CD4^+^ T cells were identified, but a number of these had overlapping characteristics.	CyTOF, scRNA‐seq, mfIHC

## AUTHOR CONTRIBUTIONS

**Sooin Byeon:** Conceptualization (equal); data curation (equal); writing – original draft (equal); writing – review and editing (equal). **Taymin du‐Toit Thompson:** Writing – original draft (equal). **Josef Gillson:** Writing – original draft (equal). **Anthony J Gill:** Writing – review and editing (equal). **Jaswinder S. Samra:** Writing – review and editing (equal). **Anubhav Mittal:** Conceptualization (equal); supervision (equal); writing – review and editing (equal). **Sumit Sahni:** Conceptualization (equal); supervision (equal); writing – review and editing (equal).

## FUNDING INFORMATION

No funding was available for this project.

## CONFLICT OF INTEREST STATEMENT

No conflicts of interest were declared by the authors.

## Data Availability

Not applicable.
